# Psychiatry

**Published:** 1904-11-26

**Authors:** 


### Progress in Medicine and Surgery.

### PSYCHIATRY.

(*Continued from page* 143.)

**Manic-Depressive Insanity. — Prognosis. —** The duration of attacks of manic-depressive insanity is variable. Those cases which run their entire course as the maniacal phase, or with alternating maniacal and depressed periods, have an average duration of about twelve to eighteen months (recurrent mania). Those cases presenting the purely depressive symptoms run a somewhat shorter course of from two to six months. The prognosis is good as regards recovery from a single attack especially at the earlier attacks of the disease, but as regards permanent recovery the prognosis is absolutely bad, for recurrence will be noted with certainty before long (within a few years).

**Diagnosis.**—The history of previous attacks corresponding to the depressive or maniacal phases of a present attack will help to determine the diagnosis. Care, however, must be taken not to confound this disease with dementia præcox, or with the involutional melancholia of middle age (forty years of age and later). The maniacal form can be distinguished from general paralysis by the characteristic physical signs peculiar to the latter.

**Intermittent or Periodic Melancholia.**—A. Gordon[3] of the Jefferson Medical College, Philadelphia, points out that those insanities which are essentially marked by a strong tendency to periodic recurrence, are not infrequently seen in private life, since such patients are, in the intervals between their attacks, very often lucid and rational in mind and conduct and capable of managing their own affairs. Hence they are not sequestrated in asylums. Alienists of a generation past maintained that the intervals between the attacks must be of equal duration in order that the disease be called periodic insanity, but further study has shown that this need not be so. Mania is more likely to occur periodically than melancholia, while circular insanity has shorter intervals of immunity. A record is given of three cases observed during the past four years. The first was that of a woman aged 35 years. Four years ago, after her fourth confinement, she grew depressed, lost interest in her work and in her family, and became the victim of sad and depressing ideas. She gradually got worse, lost her appetite, and wasted away. Symptoms of headache, partial insomnia, frightful dreams and visual hallucinations were present. This mental state continued for two months, except for slight occasional remissions lasting only a few hours. One morning the patient awoke in a calm and peaceful manner, called her children, began to caress them, and offered to prepare breakfast for the family. The mental faculties became clear, she began to take an interest in her surroundings and in herself, and the melancholic state entirely disappeared. She became pregnant again and was normally confined. Three weeks after the confinement and without any preliminary symptom, the old mental trouble returned. The second attack had the same features as the first, lasted the same length of time, and terminated as abruptly. A subsequent pregnancy occurred. Six weeks after the confinement the old trouble returned. Without any prodromal symptom she became markedly depressed, with delusions of unworthiness, painful depression of spirits, and slight longing to suicidal acts. The disease passed through the same phases as in the two previous attacks, with the exception that the duration on this occasion was four months. The termination in recovery was abrupt. "The patient is now perfectly well; a year has elapsed since the last attack." She was always considered a girl of highly irritable disposition and she suffered from "obsessions" at the age of 16 years. The other two cases were also females. "The evolution of the disease, as well as other features, are so different from the common type of melancholia that they deserve a special mention. . . . In all of them there were no morbid symptoms immediately preceding the onset of each attack. While the ordinary melancholia develops in an insidious and progressive manner. . . . . in the periodic melancholia of the intermittent type the onset is strikingly sudden." Finally, it is noted that the state of mind of the patients between the individual attacks is one of comparative mental lucidity. A hereditary tendency to brain disease is strongly marked in cases of periodic melancholia. This indicates a degenerate cerebral constitution, one which predisposes to repeated attacks of insanity. The author states that his conclusions are in accord with those of Krafft-Ebing in these respects.

[5] Journ. of Ment. Sci., Oct. 1904.

*(To be continued.)*

